# The summer heatwave in 2022 and its role in changing permafrost and periglacial conditions at a historic mountain pass in the Eastern Alps (Hochtor, Hohe Tauern Range, Austria)

**DOI:** 10.1002/ppp.2205

**Published:** 2023-08-26

**Authors:** Andreas Kellerer‐Pirklbauer, Julia Eulenstein

**Affiliations:** ^1^ Cascade – The Mountain Processes and Mountain Hazards Group, Institute of Geography and Regional Science University of Graz Graz Austria

**Keywords:** alpine cryosphere, ground temperature monitoring, heatwave summer 2022, historical data, long‐term permafrost degradation, repeated ERT measurements

## Abstract

Air temperatures in Europe in 2022 had been the highest on record for the meteorological summer season [June, July and August (JJA)], with +1.3°C above the 1991–2020 average. We studied the effects of recent warming on permafrost and periglacial conditions at a historical mountain pass in the Eastern Alps (Hochtor, 2,576 m asl, 47.08°N, 12.84°E). We used ground temperature data (2010–2022), repeated electrical resistivity tomography measurements (2019, 2022) and auxiliary data dating back to Roman times. We quantified permafrost conditions, evaluated frost‐related weathering and slope processes and assessed the impact of atmospheric warming on it. Results show that summer ground surface temperatures increased by 2.5°C between 1891–1920 and 1991–2020, whereas frost‐related weathering and periglacial processes decreased. The summers of 2003, 2015, 2019 and 2022 were the four warmest ones in 1887–2022. Hochtor changed in 2010–2022 from an active permafrost site to an inactive one with supra‐permafrost talik. A general three‐layer structure was quantified for all three ERT profiles measured. The middle, 5–10 m thick layer is ice‐poor permafrost detected in 2019, whose existence, although smaller, was confirmed in 2022. Resistivity decreased at the three profiles by 3.9% to 5.2% per year, suggesting permafrost degradation. We interpret the resistivity changes between the summers of 2019 and 2022 as a long‐term signal of permafrost degradation and not as the single effect of the summer heatwave in 2022. As our data show how rapidly permafrost degrades and as we face an even warmer climate for the remaining part of the 21st century, we expect that near‐surface permafrost at the Hochtor site will soon be history.

## 1 INTRODUCTION

**FIGURE 1:**
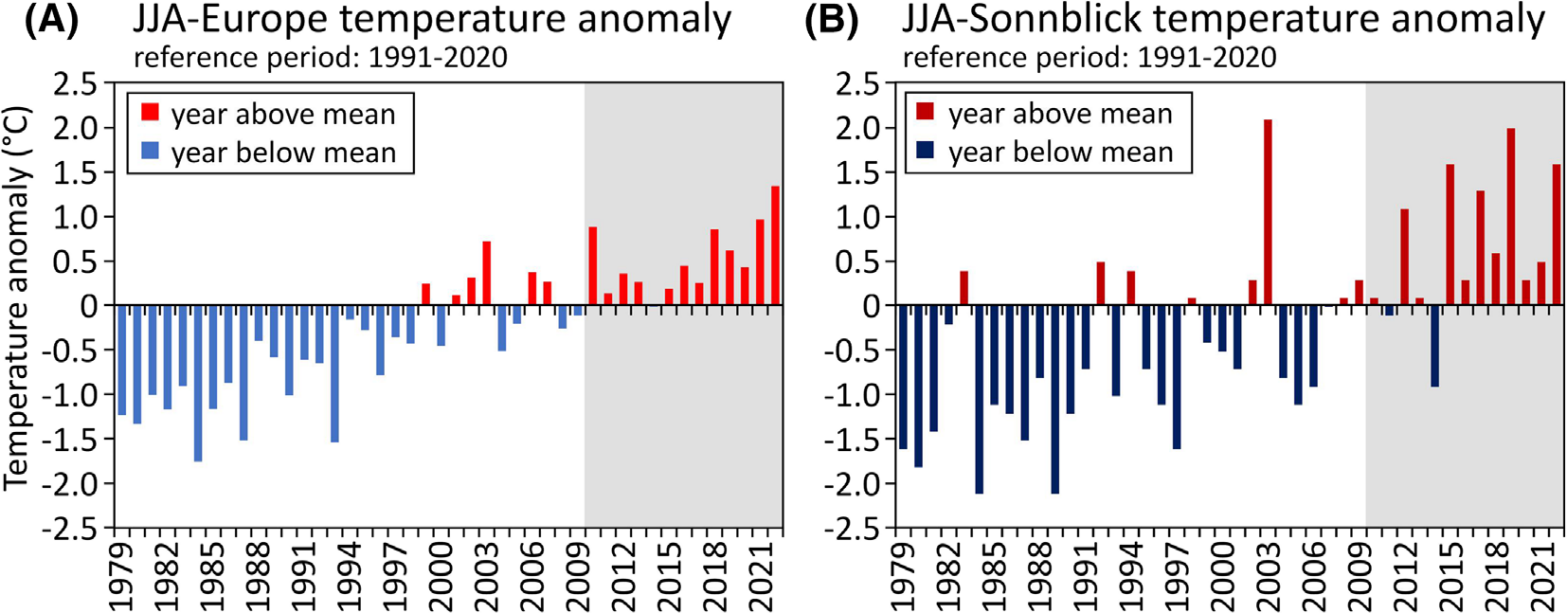
Air temperature anomaly for summer [June–July–August (JJA)] for the period 1979 to 2022 based on the reference period 1991–2020 for (A) Europe and (B) the meteorological station Hoher Sonnblick. Data sources: for Europe ERA5 (Credit: C3S/ECMWF); for Sonnblick HISTALP (www.zamg.ac.at/histalp ^2^). For the location of Hoher Sonnblick, see Figure 2. The period covered by ground temperature monitoring at the Hochtor site is indicated in grey.

Surface air temperatures in Europe in 2022 had been the highest on record for both the month of August and the (boreal) summer season (June–August) as a whole.^1^ Surface air temperatures were predominantly above the 1991–2020 average, especially in the south‐west and far north‐east of the continent. The summer that France experienced in 2022 was second only to 2003 in terms of average temperature, although the number of days that were classified as being part of a heatwave was higher in 2022 than in 2003. England had one of its hottest summers in terms of mean temperature (on par with 2018). Surface air temperatures in Europe from June to August 2022 were 1.3°C higher than the average of the reference period 1991–2020 (Figure 1A), whereas that in August itself was 1.7°C above the average.^1^ These values put summer 2022 in Europe well within the temperature range at which the Paris Agreement on climate change seeks to limit global warming.

**FIGURE 2:**
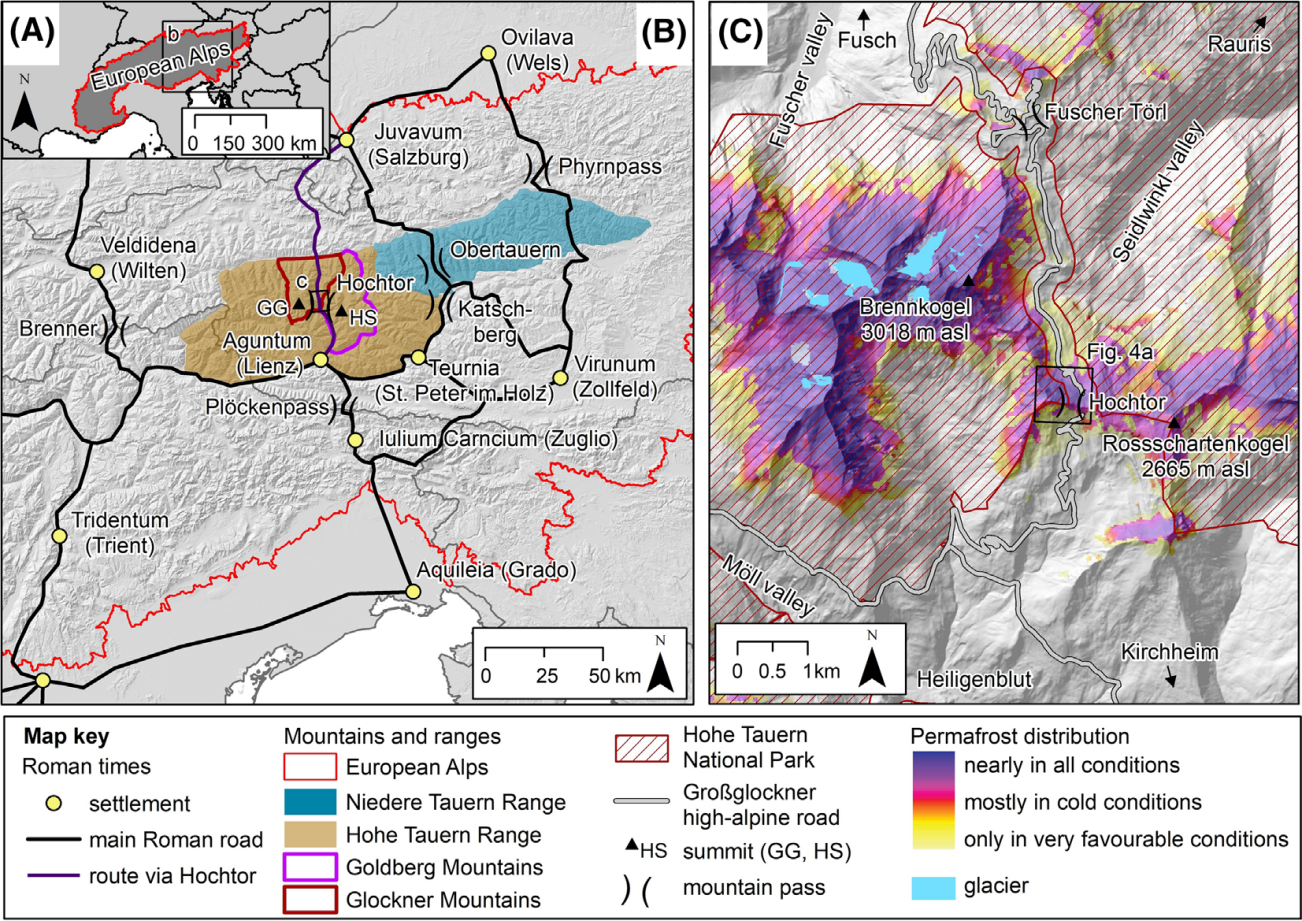
Location of the Hochtor mountain pass in the Eastern Alps and its former and present significance as an Alpine crossing: (A) overview map; (B) location of Hochtor at the main alpine drainage divide linking two subunits of the Hohe Tauern Range (Goldberg and Glockner Mountains) with important roads between the northern Adriatic Sea and the Northern Alpine foreland during the age of the Roman empire (based on figure 4 in Kandutsch^5^); (C) detailed map of the region around the Hochtor including permafrost and glacier extent. Summits: GG, Großglockner; HS, Hoher Sonnblick. Sites mentioned in the text are shown. Data sources: ESA (topography in B); Alpine convention (perimeter of European Alps); KAGIS (topography in C); permafrost^6^; glacier.^7^

High summer temperatures have a major impact on the cryosphere, for instance, during the unusually warm summer 2003 hitting the European Alps.^8^ Temperatures measured in alpine Austria in summer 2003 were even higher than the ones of 2022, as revealed by air temperature data of the meteorological station at Hoher Sonnblick at 3,106 m asl (Figure 1B; for location, see Figure 2A). Due to these high temperatures in 2003, by mid‐August, Austria's glaciers were almost entirely free of snow from the previous winter, thus favouring strong glacier ice ablation over most of their area.^9^, ^10^ The unusual summer heatwave in 2003 also impacted permafrost conditions negatively. The observed permafrost warming and degradation (i.e., complete thaw of permafrost ice), together with an increasing depth of the active layer, resulted in the mechanical weakening of the ground, leading to increased rockfall activity due to the heatwave in 2003.^11^

In this paper, we consider the results of an ongoing long‐term permafrost monitoring programme accomplished at several mountain sites in the Hohe Tauern Range, Austria (Figure 2A), focusing here on one specific site. The site of relevance of this paper is a mountain pass named Hochtor (2,576 m asl, 47.08°N, 12.84°E), which is known for its prehistoric to present significance as an often‐travelled high mountain pass.^12^ The historical importance is reflected in a variety of different sources. Some of them also provide information on periglacial and permafrost conditions. Therefore, we were able to work on the research question of how the recent past and particularly the summer of 2022 affected changes in the ground thermal regime – and subsequently on permafrost and periglacial conditions – of this site. Specifically, the aims of this study were: (i) to analyse and discuss ground temperature (GT) and permafrost conditions and potential trends in the Hochtor area during the period 2010–2022, (ii) to evaluate changes in potential frost‐related weathering during the same period and (iii) to assess the impact of the recent atmospheric warming including the summer of 2022 on the ground thermal conditions since the late 19th century. In doing so, we used direct GT data (2010–2022), repeated geophysical measurements (2019 and 2022) and auxiliary data dating back to 1887 (instrumental data) or even to Roman times (knowledge related to archaeological finds).

## 2 STUDY AREA HOCHTOR

### 2.1 Geographical setting

The Hochtor pass is in the Tauern Range. The pass connects the Seidlwinkl Valley and Ferleiten Valley located north of the pass with the Möll Valley to the south of it (Figure 2). The Tauern Range is an extensive mountain system in the central part of the Eastern Alps, covering 9,500 km^2^ in Austria and Italy (Figure 2A). The range is situated between 46°44′–47°33′N and 11°57′–15°00′E. The name ‘Tauern’ originally referred to highly located passages in the Central Alps of Austria. Later, the name came to be applied to the mountains themselves. The Tauern Range is commonly separated into the Hohe (‘high’ in German) and the Niedere (‘lower’) Tauern Range. The former covers approximately 6,000 km^2^ and reaches Mt. Großglockner at an elevation of 3,798 m asl (the highest summit of Austria). The Hohe Tauern Range consists of several subunits, of which two (Glockner and Goldberg Mountains) are of relevance for this study as the Hochtor pass connects them at the main divide (Figure 2B).

**FIGURE 3:**
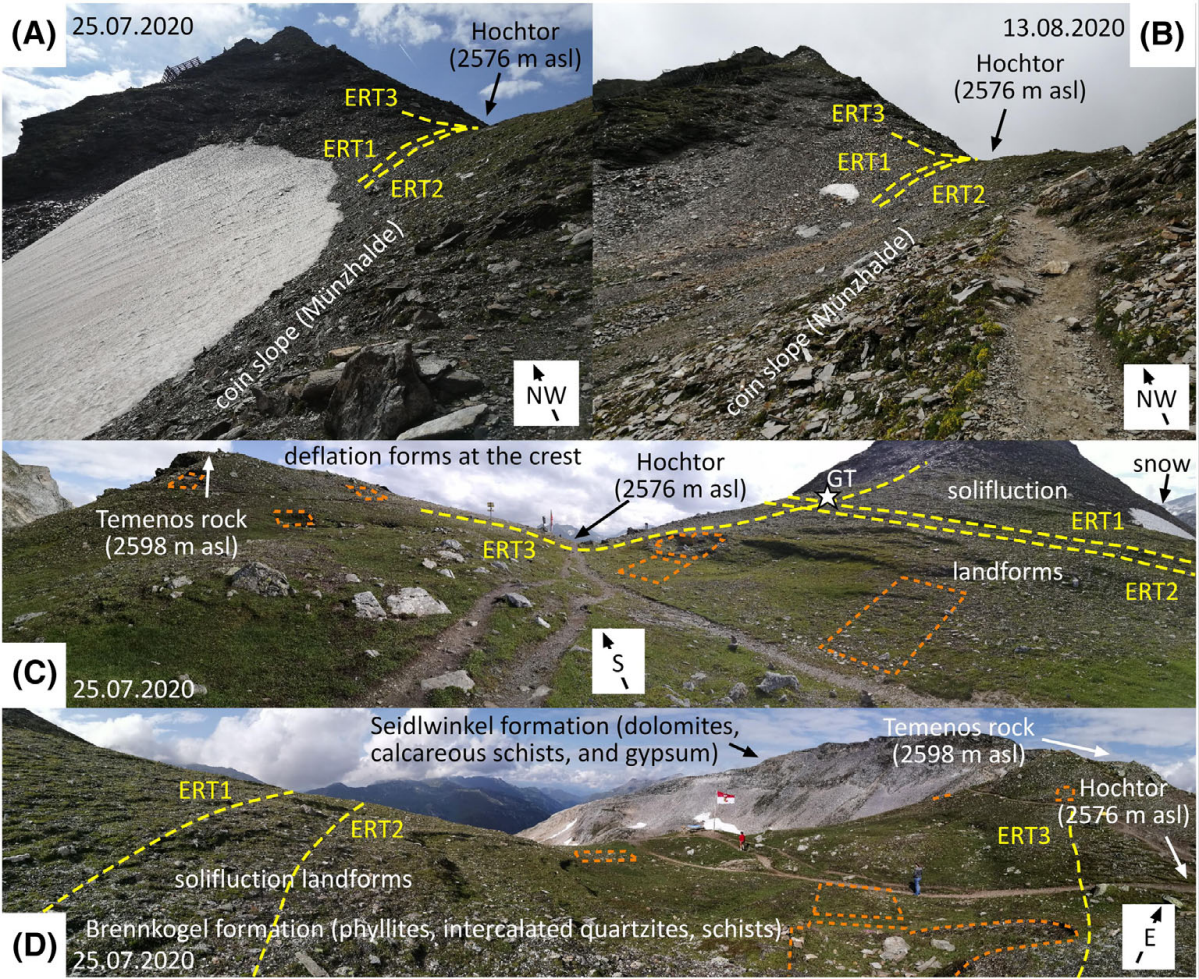
Terrestrial impressions of the study area Hochtor mountain pass: (A) and (B) show the snow cover distribution at the steep south‐facing slope of the mountain pass at the end of July and in mid‐August in 2020. At the ‘Münzhalde’ site (German for coin slope) archaeologists found different types of coins related to offerings (view towards north‐west); (C) and (D) the gentle‐sloping northern side of the Hochtor area with solifluction landforms and a hilltop east of the mountain pass where in pre‐Christian times a sanctuary was located (Temenos rock); general bedrock geology of the area is indicated. Yellow dashed lines trace the course of measured ERT profiles. Orange dashed polygons indicate excavation sites where archaeological research has been accomplished in 1995^3^, ^4^ (cf. Figure 4). Photographs by authors.

The climate at Hochtor is high alpine with a mean annual air temperature (MAAT) of about −3°C and annual precipitation of about 2,000 mm. The study area is commonly covered by snow from November to at least June^13^, ^14^ with snow patches close to the mountain pass at both the north‐facing and south‐facing slopes (partly as snow cornice) until mid‐August or even perennial (Figure 3A,B, ^15^). The vegetation at this site consists mainly of alpine grasslands, cushion plants and scree plants^16^ influenced in its growing by deflation processes at the crest (Figure 3C,D).

### 2.2 Geological and geomorphological setting

The Tauern window is the most prominent occurrence of Penninic rocks within the Eastern Alps and dominates the western half of the Tauern Range. This geological window comprises a crystalline basement (mainly granites and migmatites) and Palaeozoic to Early Tertiary meta‐sedimentary series.^17^, ^18^, ^19^ The eastern part of the Hochtor area is characterised by sporadic karst landforms related to the occurrence of dolomites, calcareous schists and gypsum of Permomesozoic age (Seidlwinkel formation^20^, ^21^, ^22^). The geology of the central and western parts of the Hochtor area (study area) is built up by the Brennkogel formation (Cretaceous), which are meta‐sediments consisting of dark, carbonate‐free to carbonate‐poor phyllites with intercalated carbonate quartzites and dark carbonate phyllites and schists.^23^

Rock breakdown in alpine environments produces commonly angular, frost‐shattered detritus. Such mountain‐top detritus is widespread in the Alps, forming autochthonous diamicts or block fields, depending on the lithology. Ballantyne^24^ stated that on well‐jointed rocks such as the phyllitic rocks at the Hochtor pass, mountain‐top detritus can develop within a few millennia of exposure to periglacial weathering.

**FIGURE 4:**
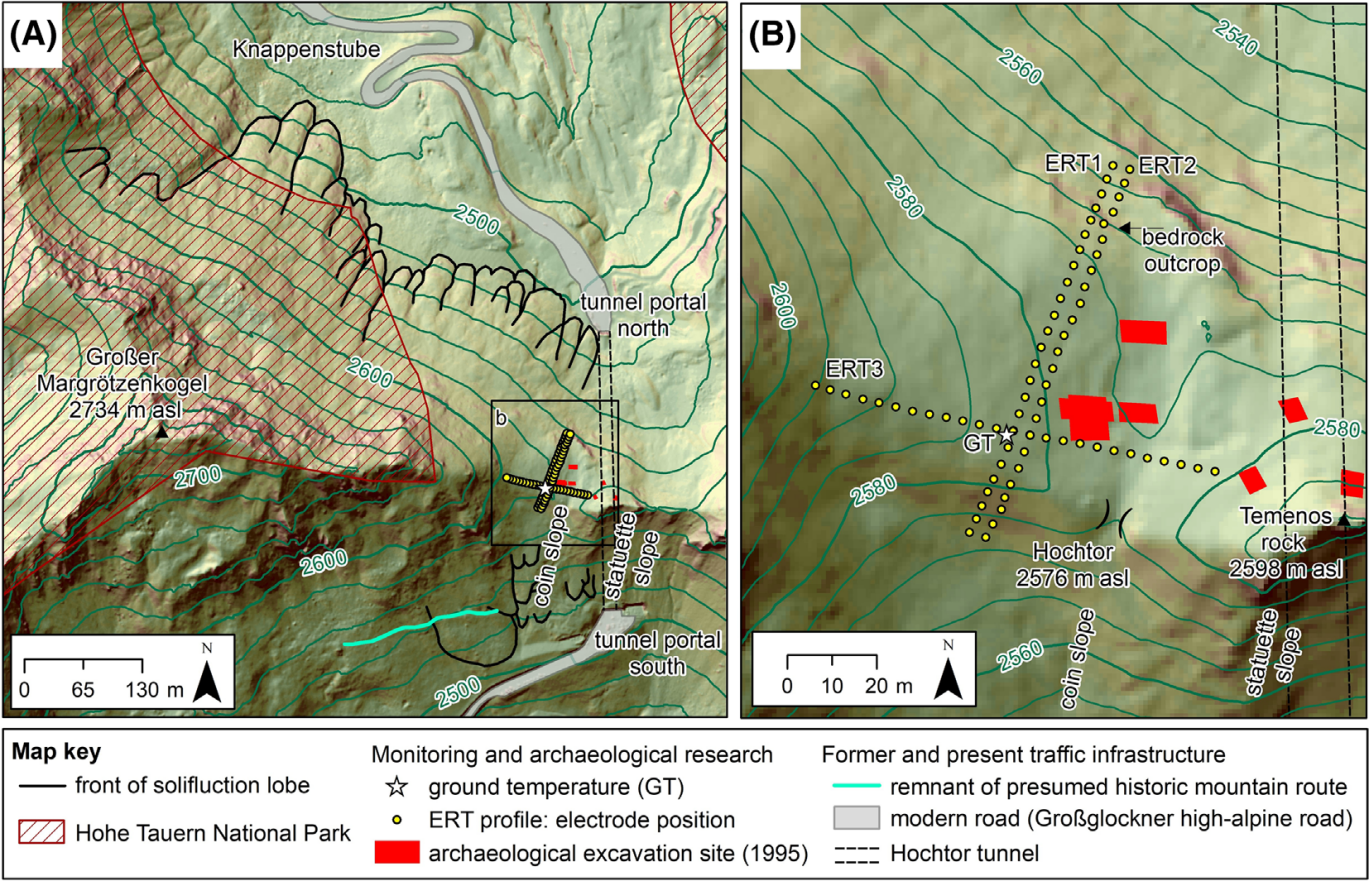
Map of the Hochtor mountain pass: (A) overview with the distribution of solifluction lobes and the course of the modern road including the Hochtor tunnel; (B) detailed map with the location of ground temperature monitoring site, the course of the three ERT profiles measured in 2019 and repeated in 2022 and the location of earlier archaeological excavation sites. The place names Temenos rock (‘Temenosfelsen’ in German), coin slope (‘Münzhalde’) and statuette slope (‘Statuettenhalde’) are informal names used by archaeologists. Data sources: KAGIS and SAGIS (topography); Hohe Tauern National Park authority (extent of the national park); Kastler^3^ and Harl^4^ (excavation sites).

Based on the regional permafrost model Alpine Permafrost Index Map (APIM), which shows an index of the estimated likelihood of permafrost occurrence for the entire Alps,^6^ permafrost is supposed to be in nearly all conditions at the mountain pass area (Figure 2C). The broad, asymmetric (steep south‐facing slope – maximum 40°; gently north‐facing slope – 5–20°) crest part of the Hochtor (Figure 3) is affected by deflation processes related to an absence of a protective winter snow cover and winds at the crest‐forming snow cornices on the lee side (Figure 3A,B). On the gentle east‐, north‐ and west‐facing slopes of the Hochtor pass, solifluction processes^25^ formed small terraces. Solifluction processes on the steeper south‐facing slope caused the dislocation of prehistoric and historic evidence (i.e., coins, statuettes – cf. below) of the former usage of the mountain pass.^4^ Turf‐banked and stone‐banked solifluction lobes are distinct landforms on both sides of the Hochtor pass. These landforms are considered active. At the nearby Fallbichl site (1.8 km to the south of Hochtor), surface velocity rates of 3.5 cm/year have been measured at a large turf‐banked lobe.^26^ The former track of Roman age over the pass was destroyed at solifluction‐affected areas on both sides of the mountain pass,^15^ particularly north‐west of the Hochtor near the Knappenstube area where up to 50 m wide and more than 100 m long solifluction lobes dominate the slope morphology (Figure 4).

### 2.3 Historical significance

The ‘hideous Alps’ – as judged by the Roman historian Livius (59 BC–AD 17) reported in Gärtner^27^ – are sometimes shown as insurmountable in ancient maps. But they were not even in early history. This also applies to the Hochtor pass. Bronze finds along the (presumed) former route suggest that this mountain crossing of the Alps was already used before antiquity in prehistoric times.^12^ Its importance probably increased with the growing relevance of the Reichenhall salt mines: one of the two routes after passing the Hochtor leads directly to Reichenhall via the Fuscher Valley (Figure 2C). Most likely, the crossing over the Hochtor was primarily reserved for the trade in goods (salt, copper, wine and lead).^28^, ^29^ Apart from trading, further evidence shows that military expeditions in Roman imperial times and pilgrims also used this route.^29^, ^30^, ^31^

The advantages of the Hochtor crossing were its shortness in alpine terrain (ideally only 1 day^12^), the mostly given passability even during winter months (little avalanche prone, marker poles), few tollbooths and low pay for the transporters.^29^ Nevertheless, a pass crossing was not without risk. Sudden cold spells or snow even in summer could end deadly.^30^ Reaching the highest point was therefore a reason for gratitude. In pre‐Christian times there was a sanctuary at the Hochtor pass, presumably at the eastern side of the pass at the nowadays informally called Temenos rock (‘Temenos’ in ancient Greek means sanctuary). The sanctuary was probably dedicated to the goodness of Jupiter.^4^, ^12^ Many coins were found at the ‘Münzhalde’ (German for coin slope) just south of the mountain pass (Figure 3A,B). Little statuettes and pieces of them were particularly excavated at the steep slope named ‘Statuettenhalde’ (German for statuette slope) south of Temenos rock.^4^, ^15^ Remains of historical pass shrines have not been found despite widespread archaeological excavation campaigns at the pass area (cf. Figures 3 and 4, ^3^, ^4^). The offerings (statuettes, coins) were also not in the assumed original place. Related to mass transport processes including solifluction,^32^ these remains of human activity were transported down the valley (Figures 3 and 4).

The decline of the importance of the Hochtor passage in historic times began in the last decade of the 16th century. This had infrastructural reasons. The pass was not passable with waggons until the construction of the Großglockner high alpine road in the 1930s.^15^ Goods had to be transported by porters or pack animals, and travellers could ride only on horseback.^29^, ^33^ This became a decisive disadvantage when the alternative Katschberg–Radstadt route was expanded for vehicles around AD 1519. Around 1760, the trade across the Hochtor probably ended.^29^ In the centuries that followed, the Hochtor mountain crossing fell into oblivion outside of the local world. There were even rumours in the 1970s when a Roman statuette (known as the ‘Hercules of the Hochtor’ dating to the first century BC^28^) was found in 1933 during the construction of the Großglockner high alpine road that it was placed deliberately by Franz Wallack – chief engineer of the Großglockner high alpine road – or his workers.^5^, ^34^ Romans travelling across the Hochtor pass sounded so unlikely at that time. It is now generally accepted that the statuette was not intentionally placed where it was found in 1933. Wallack himself emphasised the existence of the old passage and its reuse whenever possible to save construction costs.^35^

### 2.4 Present significance

Most people today do not pass the Hochtor pass for economic reasons. The Großglockner high alpine road, built in 1930–1935,^15^ was and still is used almost exclusively for scenic driving pleasure.^36^ The route connects the two federal provinces Salzburg (north) and Carinthia (south) and was chosen to highlight the scenery including the largest glacier in (Pasterze Glacier, ca. 15 km^2^) and the highest mountain of (Großglockner, 3,798 m asl) Austria.^37^ Since 1981, the Hohe Tauern National Park flanks the course of the road on both sides of the Hochtor pass (Figure 2C). Basic foundations for these developments were laid in the 18th century, when the crucial turning point in people's perception of the Alps from hideous to beautiful occurred.^38^

At Hochtor, the modern high alpine road does not follow the old track. The pass was tunnelled in 1933 and is located c.70 m above the 302 m long tunnel of the present‐day road. The tunnel reaches its maximum elevation at the south portal at 2,504 m asl. The tunnel construction was made to avoid long‐lasting (or even perennial) snow patches on both sides.^15^ This decision by Franz Wallack shows his knowledge of the snow cover conditions surrounding the new road. Some of his descriptions made during the construction process are even of relevance for assessing permafrost and periglacial conditions at the Hochtor area today, as we will see later in this article.

## 3 MATERIALS AND METHODS

### 3.1 GT data

GT at Hochtor has been measured since 2010 by us at different depths to assess the thermal conditions of the ground surface and near surface. A multichannel miniature temperature datalogger (MTD) with three individual PT1000 temperature sensors (Geo‐Precision, Model M‐Log6, Ettlingen, Germany) was installed at the pass location in August 2010 at an elevation of 2,582.5 m asl (GT in Figure 3C and 4B). GT was measured since then (1 h frequency), forming an almost continuous data series for the period 24 August 2010 to 12 September 2022 (4,403 days). Data gaps exist only for 14 days (20 August 2013; 26 to 29 August 2013; 5 to 13 September 2013) due to technical problems in 2013. Temperature sensors were placed at the ground surface (GT0) and at 10 cm (GT10) and 60 cm (GT60) below the surface using a soil auger forming a vertical profile. The PT1000 sensor nearest to the surface was sheltered from direct solar radiation by thin platy rocks that allow rather unhampered air circulation within the voids. According to the producer, the used PT1000 temperature sensors in the MTDs have an accuracy of ±0.05°C, a range of −40°C to +100°C and high long‐term stability with a calibration drift >0.01°C/year. Raw data were checked before further processing. At the uppermost sensor (GT0), a notable winter snow cover caused a recognisable zero curtain period (ZCP) for some years. With this ZCP, it was possible to check accuracy, shift or drift in the raw data as such a period is characterised by a constant temperature of 0°C. Our quality check revealed neither a distinct drift nor notable data inaccuracies.

Several parameters derived from the temperature data were calculated on a monthly, seasonally or yearly timescale (Table 1): mean ground temperature (MGT0, MGT10, MGT60), sum of positive (thawing) degree days (TDD; alternative term: ground thawing index), sum of negative (freezing) degree days (FDD; alternative term: ground freezing index), number of diurnal freeze–thaw cycles (FTC), number of frost‐free days (FFD) and number of ice days (ID) (i.e., highest temperature per day ≤0°C). Furthermore, the number of seasonal snow cover days (SCD) was estimated for the GST sensor by calculating the sum of days with a notable snow cover damping effect, considering as a threshold a weekly standard deviation (SD) of the mean daily ground surface temperature of ≤0.25°C.^39^ In addition, the number of days with mean temperatures exceeding certain thresholds (between +5°C and −15°C with 5°C intervals) was calculated for each sensor.

**TABLE 1:** List of calculated parameters based on ground temperature data.

Type	Parameter		Relevant sensor (code)			Unit
	Code	Description (timescale)	0 cm (GT0)	10 cm (GT10)	60 cm (GT60)	
Thermal characteristics	MGT	Mean ground temperature (m, s, y)	X	X	X	°C
	TDD	Thawing (positive) degree days (y)	X	X	X	Sum +°C
	FDD	Freezing (negative) degree days (y)	X	X	X	Sum − °C
	FTC	Days with diurnal freeze–thaw cycle (y)	X	X	X	Number
	FFD	Frost‐free days (y)	X	X	X	Number
	ID	Ice days (y)	X	X	X	Number
	T(x)	Number of days with mean temperature below (−15°C, −10°C, −5°C, 0°C) or above (0°C, 5°C) certain values (s, y)	X	X	X	Number
	FCW36	Days in frost‐cracking window −3°C to −6°C (y)	X	X	X	Number
	FCW38	Days in frost‐cracking window −3°C to −8°C (y)	X	X	X	Number
Seasonal snow cover	SCD	Snow cover duration (y)	X	—	—	Number

*Note*: m, month; s, season (meteorological seasons DJF, MAM, JJA, SON); y, year (monitoring year: 1 September to 31 August of the following year).

To assess the severity of freezing events, the number of days when a given sensor was within a certain temperature range below freezing [‘frost‐cracking window’ (FCW)] was calculated.^40^ A day within the FCW implies that during a given day temperatures between −3 and −6°C (FCW36) or −3°C and −8°C (FCW38), respectively, were measured (cf. Figure 5 in Hallet et al. 1991 for the two temperature ranges). The used thresholds for defining the FCWs are subjective because of the laboratory‐origin and sandstone‐specific values for frost cracking used by Hallet and others. FCW values over a 12‐year period are nonetheless useful to investigate the climate‐change impact on frost‐related weathering.

**FIGURE 5:**
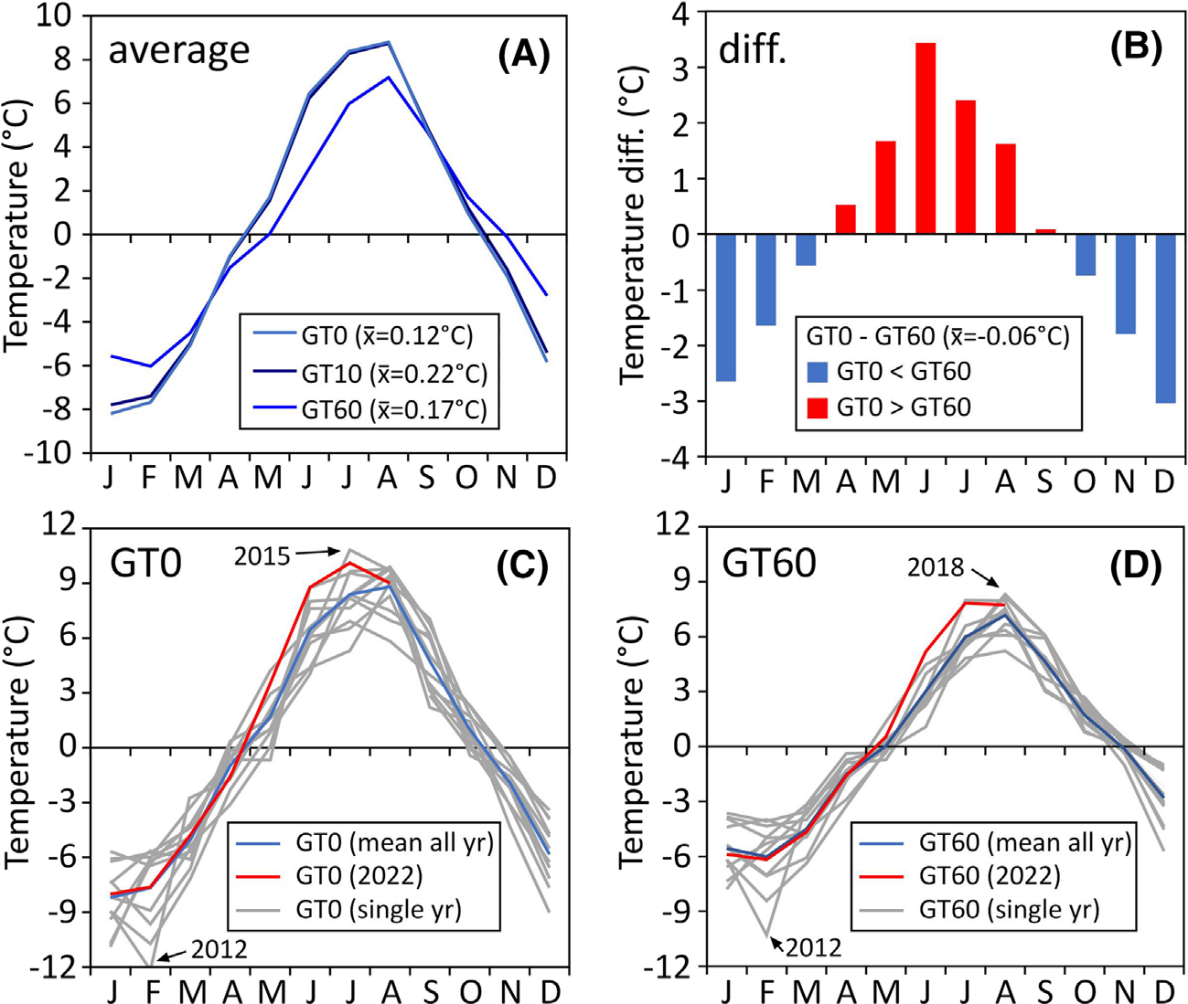
Ground thermal conditions at site GT based on monthly data from September 2010 to August 2022: (A) average mean monthly temperatures at the surface (GT0) as well as at 10 (GT10) and 60 cm (GT60) depths; (B) average temperature difference between the sensors at the surface (GT0) and at 60 cm depth (GT60); monthly values for individual years with focus on the mean‐ and 2022‐values for GT0 (C) and GT60 (D).

### 3.2 Electrical resistivity tomography

Knowledge of subsurface electrical resistivity conditions allows the distinction between unfrozen and frozen material as well as massive sedimentary ice.^41^, ^42^ Electrical resistivity is a physical parameter related to the chemical composition of a material and its porosity, water and ice contents as well as temperature.^43^ Two‐dimensional electrical resistivity tomography (ERT) uses multielectrode systems and two‐dimensional data inversion to receive a resistivity model of the subsurface.^42^

ERT was applied at three profiles (ERT1‐3) at the pass location in 2019 (Figures 3 and 4; Table 2). ERT measurements at the same profiles were repeated in 2022. Vegetation along the ERT profiles was mostly patchy (partly related to deflation) with discontinuous alpine grassland as well as cushion and scree plants (Figure 3). ERT1 and ERT2 are both oriented S–N, whereas ERT3 proceeds from E to W intersecting the other two profiles at profilemeter 44 (ERT2) and 48 (ERT1). ERT1 and ERT2 run parallel shifted by 4 m. The material along the profiles is characterised by the debris of different grain sizes with a dominance of fines. Around electrode no. 21 (EL21) at profilemeter 80 of both ERT1 and ERT2, loose rocks of up to boulder size and weathered, platy foliated bedrock related to a bedrock outcrop (see Figure 4B) reduced the penetration depth of the spike electrodes into the ground. At one electrode (ERT2, EL21), a jumper cable connection was necessary.

**TABLE 2:** Overview of ERT profiles at the Hochtor site measured in 2019 and 2022.

Code	Dates (DD/MM/YY)	Elevation (m asl)		Course	Setup (LE/SP/EL)	Array	Annotation
		Minimum	Maximum				
ERT1	17/08/2019, 12/09/2022	2,562.5	2,582.4	S‐N	96 m/4 m/25	W, S	Parallel to ERT2; rock outcrop at EL21 to EL22
ERT2	17/08/2019, 12/09/2022	2,561.7	2,581.4	S‐N	96 m/4 m/25	W, S	Parallel to ERT1; rock outcrop at EL21
ERT3	17/08/2019, 12/09/2022	2,575.3	2,594.8	E‐W	96 m/4 m/25	W, S	ERT3 crosses ERT2 (at EL12) and ERT1 (at EL13)

*Note*: asl, above sea level; EL, number of electrodes; LE, length of profile (m); S, Schlumberger; SP, spacing of electrodes (m); W, Wenner. For location, see Figures 2 and 3.

During both geophysical campaigns (17 August 2019 and 12 September 2022), a GeoTom‐2D system (Geolog2000 ‐the spike electrodes are also manufactured by the Geolog company, Augsburg, Germany) with one multicore cable was used with 25 take‐outs, 25 spike electrodes (stainless steel), an electrode spacing of 4 m and thus a total profile length of 96 m. We applied both the Wenner and the Schlumberger arrays although the focus here is given on the Wenner data, which are more suitable for layered structures.^42^ A real‐time kinematic (RTK) global navigation satellite system (GNSS) was utilised to measure the position of each electrode and thus the course of the three profiles (Figure 4B). For this, we used a Topcon HiPer V differential GPS system and obtained correction signals from a national correction‐data provider (EPOSA, Augsburg, Germany).

The apparent electrical resistivity data were two‐dimensionally inverted in the software RES2DINV using the robust inversion modelling approach.^44^ ERT data were checked before processing for abnormally low or high‐resistivity values commonly related to measurement errors and/or bad electrode contact usually visible at all depths of the ERT profiles. Such bad data points were excluded as suggested by Kneisel and Hauck.^42^ The number of iterations was five, if not otherwise stated. For studying the changes in the subsurface between 2019 and 2022, we accomplished a time‐lapse analysis for each of the three ERT profiles in RES2DINV. For this, we used our Wenner data and followed the time‐lapse resistivity inversion recommendations by Loke (Penang, Malaysia).^44^

Finally, to analyse the quality of the detected subsurface structures in the inverted ERT data, the depth of investigation (DOI) index was calculated for each geophyscial dataset. The DOI method is based on Oldenburg and Li^45^ and attempts to assess the parts of the inverse model. Those are well constrained by the data by quantifying the sensitivity of the inversion result to changes in the inversion parameters. The DOI index results were normalised. DOI values close to zero suggest reliable results, whereas values close to one indicate the opposite. As conservative threshold for reliable data 0.2 was applied (cf.^46^). The following DOI parameters were considered: first model reference factor: 0.1; second model reference factor: 10.0; optimise inversion settings: yes; damping factor for reference model: 0.05; type of background reference model: multiplication; inversion method: smooth (L2 norm); factor to extend depth range: 3.0; number of iterations: 3.^44^

### 3.3 Auxiliary data

Historic written descriptions and photographs related to the inner structure and periglacial conditions of the Hochtor area were taken from the literature (mainly from Wallack^15^) and the archive of the Großglockner Hochalpenstraßen AG (Archive GROHAG). Descriptions of the near‐surface layers based on archaeological excavations at the mountain pass (see Figure 4) and neighbouring sites are based on Harl^4^, ^32^ and Kastler.^3^ Monthly air temperature data from the high‐alpine meteorological station at Hoher Sonnblick (3,106 m asl) were taken from the HISTALP database (www.zamg.ac.at/histalp; for details see Auer et al.^2^).

Snow data (monthly values of the maximum snow depth) were also provided for this station by GeoSphere Austria for the period from January 2000 to December 2022. The Sonnblick station provides an almost continuous record of air temperature data since 1886 (annual data since 1887) and is located only 10 km south‐southeast (SSE) of the Hochtor site (Figure 2A).

## 4 RESULTS

### 4.1 Ground thermal conditions

Average mean monthly temperatures for each of the three sensor depths (GT0, GT10, GT60) covering the period September 2010 to August 2022 are plotted in Figure 5A. Average mean monthly values at the surface (GT0) vary between −8.2 (January) and 8.8°C (August) and, thus, cover a temperature range of 17.0°C. For the sensors at 10 (GT10) and 60 (GT60) cm depths, these values are −7.8 C (January), 8.7 C (August) and 16.5°C (amplitude) for the former, whereas −6.0°C (February), 7.2°C (August) and 13.2°C (amplitude) for the latter. At all three sensors, the monthly maximum is reached in August contrasting to the minimum, which occurs in January close to the surface and in February at 60 cm depth related to thermal damping and delaying. The mean monthly temperature is up to 3.4°C higher at the surface compared to 60 cm depth related to spring and early summer ground warming (Figure 5B) from the surface. During the winter half‐year, October to March, the surface sensor measured up to 3°C lower mean monthly temperatures caused by efficient winter surface cooling. On an annual mean, sensor GT10 is 0.1°C warmer compared to the surface sensor and 0.05°C warmer compared to the one at 60 cm depth. Mean annual ground temperatures (MAGT) based on calendar years range from −0.43°C to 0.61°C at GT0 and from −0.54°C to 0.59°C at GT60, respectively. The average MAGT for the entire 12‐year monitoring period and for all three sensors is between 0.1°C and 0.2°C.

Figure 5C,D (GT0 and GT60, respectively) shows the values for each month and each year with data depicting the variability in the different years. Note the exceptionally cold February 2012 (−12.7°C) contrasting to the unusually warm July 2015 (10.8°C). In 2022, temperatures at 7 out of 8 months (apart from April; −0.6°C colder) have been above average at the surface sensor GT0 with maximum anomalies in May to July with +1.7°C to +2.3°C. This high exceedance of mean monthly values was comparable at 60 cm depth (GT60) during June and July in 2022. In contrast, May and August 2022 were only slightly warmer than average at GT60.

**FIGURE 6:**
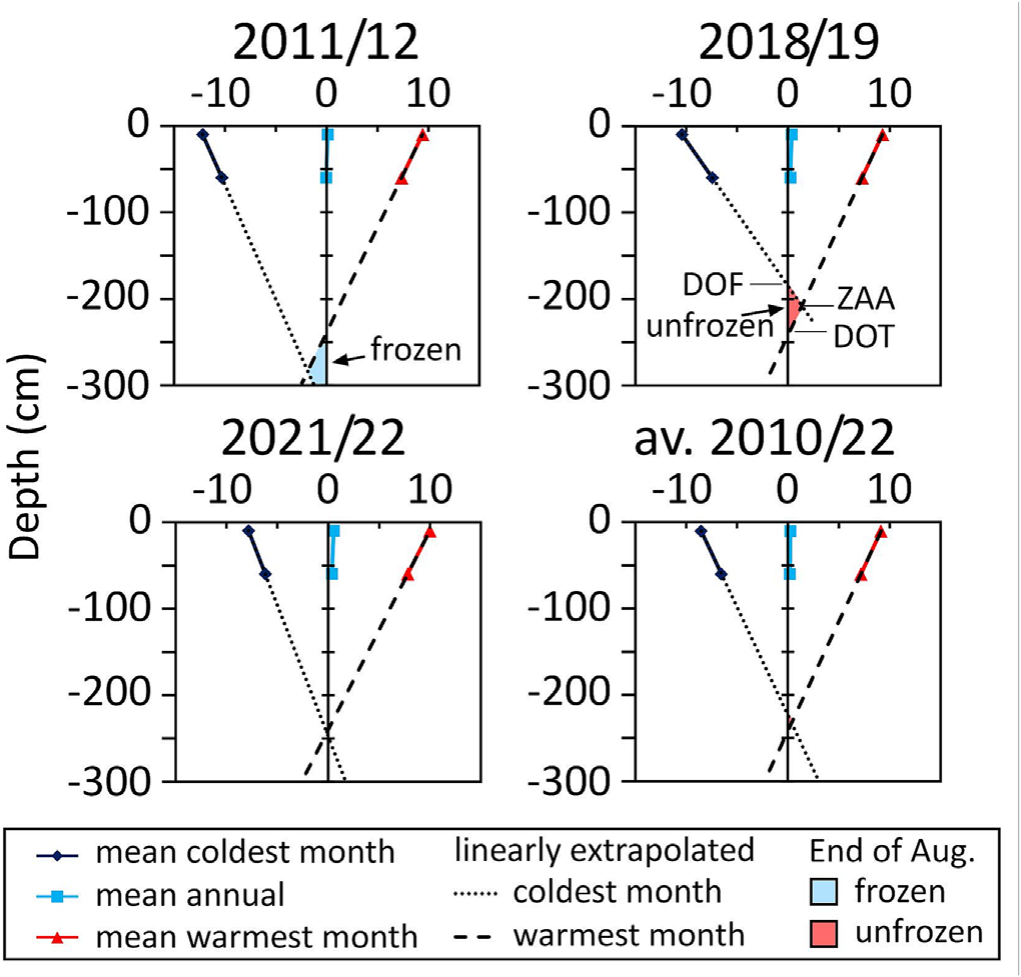
Mean temperature profiles at site GT based on monthly values of GT10 and GT60: mean annual, mean monthly minimum (mean of the coldest month) and mean monthly maximum temperatures (mean of the warmest month) for the year 2011/2012 (most permafrost‐favourable year), 2018/2019 (year with ERT campaign no. 1), 2021/2022 (ERT campaign no. 2) and the average of 2010 to 2022. Linear trends for the minimum and maximum temperatures are shown implying the depth of the zero annual amplitude, depth of freezing and depth of thawing for each period.

Figure 6 shows MGT profiles at site GT for the three 1‐year periods (in this case, ‘monitoring year’ means the period 1 September until 31 August of the subsequent year) 2011/2012, 2018/2019 and 2021/2022 as well as the average for the 12‐year period from 2010/2011 to 2021/2022 based on monthly values where the mean annual temperature, the mean of the hottest month and the mean of the coldest month are plotted against depth. The trends of the latter two tend to meet at a point where the daily and annual amplitude is about 0°C (see van Everdingen^47^). By applying this method of extrapolating temperature profiles, the depth of the zero annual amplitude as well as the depth of thawing (DOT) and freezing (DOF) can be estimated. DOT equals the active layer in cases of permafrost conditions. DOF corresponds to seasonal frost thickness in non‐permafrost environments. DOT and DOF for all 12 years are summarised in Table 3. As these trends are commonly nonlinear as shown, for example, by borehole data,^48^ applying such a linear trend gives only an estimation. Estimated annual thawing depths exceeded annual freezing depths in 7 out of 12 years during the period 2010 to 2022. In the years 2017/2018 and 2019/2020, the calculated DOT was between 120 and 125 cm deeper than the calculated DOF. In contrast, in the 5 years 2010/2011, 2011/2012, 2012/2013, 2014/2015 and – after a long gap in between – 2021/2022, freezing depth exceeded the thawing depth, although in the last year of data, this difference was only 6 cm. On average for the 12 years of data, the DOT exceeded by 17 cm the DOF. Regardless of the exact depth values of DOT and DOF, this result suggests permafrost degradation conditions at the GT site.

**TABLE 3:** Calculated depth of thawing (DOT), depth of freezing (DOF) and the difference between the two for each monitoring year (September–August) between 2010 and 2022 based on mean monthly temperatures of coldest and warmest months for each year and average.

Year	DOT (cm)	DOF (cm)	DOT − DOF (cm)
2010/2011	211	278	67
2011/2012^a^	239	333	94
2012/2013	217	269	52
2013/2014	219	175	−44
2014/2015	209	275	66
2015/2016	196	177	−19
2016/2017	272	201	−71
2017/2018	336	216	−120
2018/2019^a^	244	184	−60
2019/2020	331	206	−125
2020/2021	252	198	−54
2021/2022^a^	241	247	6
Average^a^	241	224	−17

^a^ Shown in Figure 6.

**FIGURE 7:**
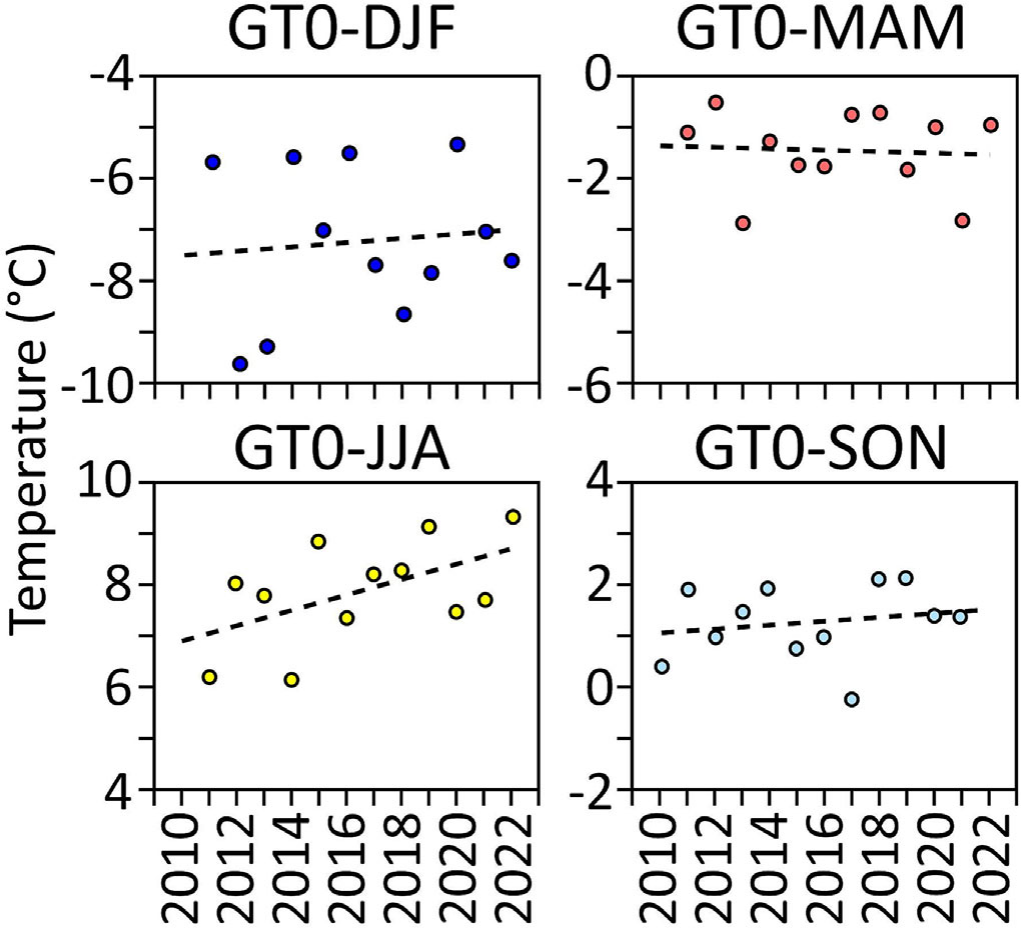
Evolution of the mean seasonal ground temperature at the uppermost sensor (GT0) at site GT based on data between autumn (SON) 2010 and summer (JJA) 2022.

The evolution of the seasonal mean temperatures for winter [December, January and February (DJF)], spring [March, April and May (MAM)], summer (JJA) and autumn [September, October and November (SON)] is depicted in Figure 7, covering the period from September 2010 to August 2022. No clear and statistically significant trends in seasonal temperature evolution have been detected for the winter, spring and autumn seasons. Only a tendency towards slight warming was revealed for DJF and SON. In contrast, rather stable conditions to even slight cooling were quantified for MAM. This apparent cooling trend in the springs 2011 to 2022 is surprising but agrees well with Sonnblick station air temperature data for the same period. This must be seen as a temporary exception. In the long term (at Sonnblick this means 1887–2022), there is a clear spring warming trend. Only the data for JJA suggest a distinct and almost significant warming trend during the 12‐year monitoring period with a p‐value very close to 0.05 (*p* = *0.07*).

Based on mean annual data of the 12 monitoring years since 2010, trends (although statistically insignificant) of an increase in the mean annual values (monitoring years) have been detected for MGT, TDD and FFD (no graphics shown). No notable trends were detected for FDD, ID and most of the number of days with mean temperatures below (−20°C, −15°C, −10°C, −5°C, <0°C) or above (≥0°C, >5°C, >10°C, >15°C) certain values. In contrast, negative trends were revealed for the diurnal FTC, days in the FCWs −3°C to −6°C (FCW36) and, respectively, −3°C to −8°C (FCW38). Days with a notable seasonal snow cover had been reduced from 18 in 2010/2011 to 0 by 2013/2014. Since then, no longer lasting snow cover was detected at site GT anymore. Statistically significant changes during the monitoring period have been calculated only for two of the parameters: the decrease in the days with a snow cover/SCD (from 18 in 2010/2011 to 0 in 2021/2022; *p* < *0.01*) and the increase in the number of days with a MGT >5°C (from 67 in 2010/2022 to 116 in 2011/2022; *p* < *0.05*). High interannual fluctuations for all parameters obscure, nevertheless, in most cases meaningful trends.

The impact of high interannual variability is reduced by averaging several years. As a compromise between data series length and number of subperiods, we divided the 12‐year data into three time slices of 4 years each. Results are summarised in Table 4 and indicate trends related to ground warming for the parameters MGT, TDD, FDD and number of days with a mean temperature below −15°C, −10°C and above 5°C. Interestingly, a weak trend for ground cooling is suggested by the parameter number of days with a mean temperature below 0°C. A decrease in the potential weathering by freeze–thaw action during the 12‐year monitoring period is revealed by the decreasing number of FTC and the number of days within the FCW for both temperature ranges (FCW36, FCW38) indicated in Table 1.

**TABLE 4:** Four‐year average values based on monitoring years (1 September to 31 August of the subsequent year) for different ground temperature parameters based on data of sensor GT0.

Four‐year period	MGT	TDD	FDD	FTC	FFD	ID	FCW36	FCW38	SCD	T < −15	T < −10	T < −5	T < 0	T ≥ 0	T > 5
2010/2011–2013/2014	−0.18	883	−954	70	123	166	24	59	10	4	18	90	196	166	79
2014/2015–2017/2018	0.14	994	−927	43	143	178	11	39	0	1	17	95	198	168	92
2018/2019–2021/2022	0.39	1,048	−895	50	140	167	11	36	0	0	15	88	203	162	102
Trend	+	+	+	−	o	o	−	−	−	−	−	o	+	o	+

*Note*: −, negative; o, none; +, positive. For abbreviations and units, see Table 1.

### 4.2 Subsurface conditions based on geophysics

**FIGURE 8:**
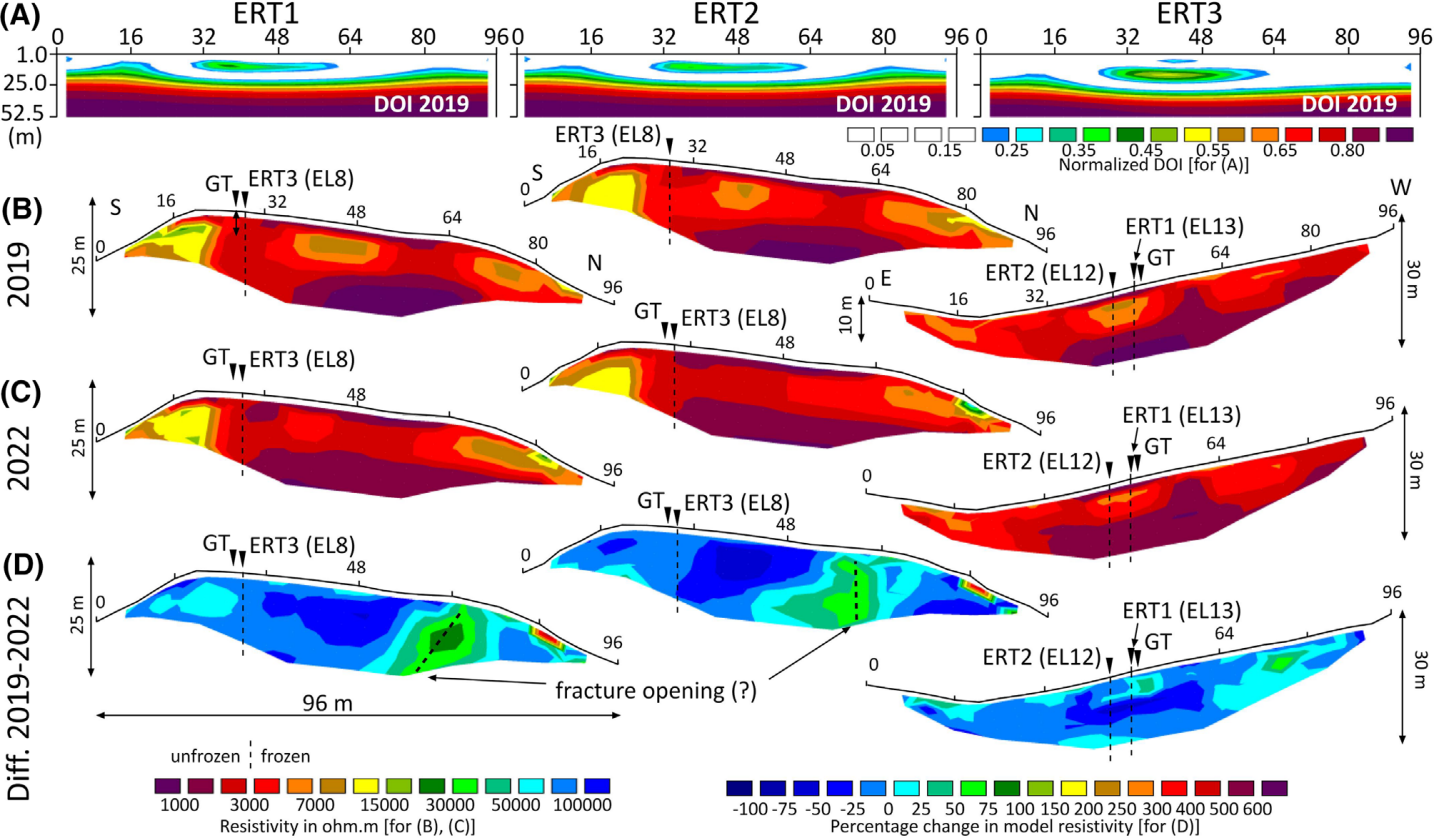
ERT results at the Hochtor area for ERT1, ERT2 and ERT3: (A) depth of investigation index for the 2019 data; white area (<0.2) indicates reliable data; (B) inversion results 2019 with topographic correction: Wenner array, five iterations, RMS errors 2.9–3.4; (C) inversion results 2022 with topographic correction: Wenner array, five iterations, RMS errors 3.2–3.8; (D) time‐lapse inversion results: negative values, decrease in resistivity; positive values, increase in resistivity. Dashed lines at the profiles indicate profile intersections.

Results of the DOI analysis show high data quality for all three profiles and for both years if the normalised threshold for reliable data of 0.2^46^ is used (Figure 8A). All three pairs of profiles of the data from 2019 and 2022 show results that are consistent with one another. In addition, ERT1 and ERT2 are very similar, with high‐quality data along most of the profile and up to the lowest measured level (ca. 16–17 m) apart from an elongated lens of poorer data quality at profilemeter 32–56 and at a depth of c. 8–11 m. At ERT3 high‐quality data were revealed for the entire profile down to c. 12 m depth. DOI results for 2019 are exemplarily depicted in Figure 8A.

**FIGURE 9:**
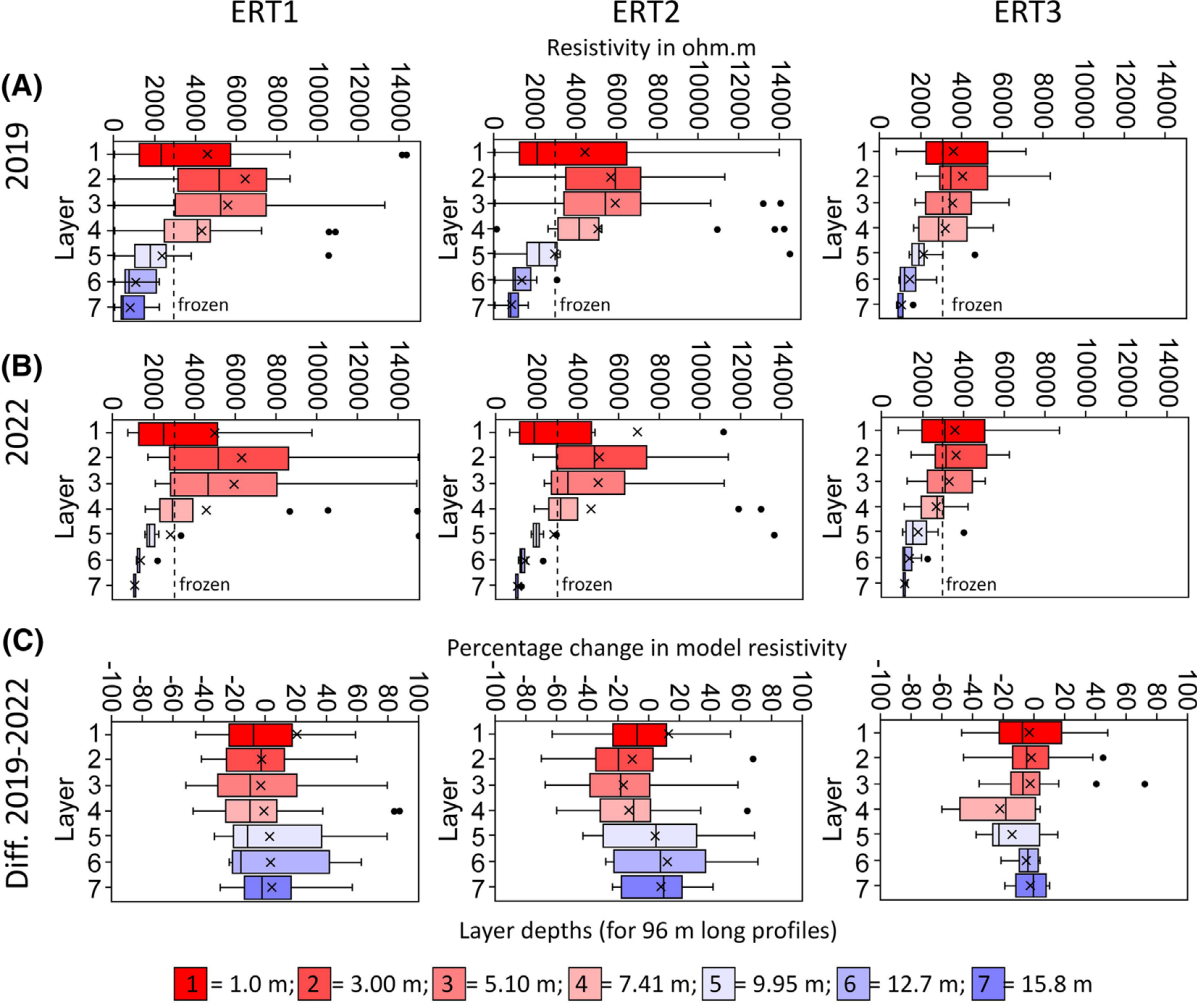
Box plots of resistivity values at the seven different layers over the entire measuring profile for ERT1, ERT2 and ERT3 (Wenner array, five iterations, all 96 m length): (A) for 2019; (B) for 2022; (C) change in resistivity between 2019 and 2022; negative values, decrease in resistivity; positive values, increase in resistivity. Layers (L) converted to depth for the 96 m long profiles. X marks the arithmetic mean. Stippled lines at 3,000 Ω.m mark the transition of unfrozen to frozen (air‐void poor) material. Resistivity values >15,000 Ω.m are not indicated in (A) and (B).

ERT results of 2019 are presented in Figure 8B. Maximum resistivity values in 2019 were 20,970 Ω.m for ERT1, 24,032 Ω.m for ERT2 and 8,335 Ω.m for ERT3. Median values were similar for the three profiles with 3,088, 3,357 and 2,840 Ω.m for profiles ERT1 to ERT3. In all three profiles, a distinct layering was discovered by geoelectrics along most of the profile lengths. An exception is the southernmost and northernmost parts of ERT1 and ERT2. Regarding the layering, an uppermost layer with a thickness of about 2–3 m comprises resistivity values of up to 2 k Ω.m. This layer is not developed over the entire profile length. Further below, a 5 to 10 m thick second layer with resistivity values of up to 10 k Ω.m is well developed. This second layer comprises lens structures in all three profiles. A third layer is detectable at sections of various lengths in all three profiles starting at about 10 m below the surface. An important exception to these layered structures is the southernmost 25 m of ERT1 and ERT2, where resistivity values exceed 10 ka Ω.m over the top 10 m (total profile thickness). Figure 9A depicts box plots of the resistivity distribution in 2019 for each of the seven different depth layers for ERT1, ERT2 and ERT3. These graphs show in a different way the layered structure with the highest medians at layers 2 (3.00 m) and 3 (5.10 m), in general, lower values (although with a high‐value range) at layer 1 (1.00 m), as well as decreasing median values with increasingly smaller ranges further below to a depth of 15.8 m.

ERT results of 2022 are presented in Figure 8C. Maximum resistivity values in 2022 were at all three profiles higher compared to 2019: 26,544 Ω.m for ERT1, 70,293 Ω.m for ERT2, and 8,704 Ω.m for ERT3. In contrast, median values in 2022 were at all three profiles lower (by 332 to 524 Ω.m) compared to 3 years earlier with 2,703, 2,834 and 2,508 Ω.m for ERT1 to ERT3. The three tomography models from 2022 look very similar to those of 2019 with comparable patterns at all three profiles. Looking at the results in more detail, however, the lenses of high‐resistivity values in layer 2, which have become smaller since 2019, are particularly noticeable. In the middle sections of the profiles of ERT1 and ERT2, for instance, resistivity values of more than 5,000 Ω.m no longer occurred in 2022. At the northernmost sections of ERT1 and ERT2, resistivity values increased also at the surface. Figure 9B depicts box plots of the resistivity distribution in 2022 for each of the seven different depth layers for ERT1, ERT2 and ERT3. These graphs support the descriptions above and are also comparable to the ERT conditions of 2019. However, a distinct difference is the larger value range at layers 2 and 3 at the first two geoelectrical profiles and the very small value range at the lowest three levels at a depth of below 10 m. In contrast, the box‐plot graphics for ERT3 for both years look almost identical.

The differences in the resistivities in 2019 and 2022 for all three profiles based on time‐lapse inversions are shown as tomography models in Figure 8D and as box‐plot graphics in Figure 9C. The tomographs indicate a general decrease in the overall resistivities along most of the profiles. However, sections with distinct resistivity increases between 2019 and 2022 have been detected around profilemeter 60 (over the entire profile depth) and 80 (only superficially) of ERT1 and ERT2. Possible interpretations for this (including a possible fracture opening) are dealt with in the discussion chapter. At ERT3, small lenses along the entire profile reveal localised resistivity increases. On average over the entire profile length and the entire depth, resistivities decreased by 12.5% for ERT1, by 15.6% for ERT2 and by 11.7% for ERT3. This change in resistivity was, however, not uniform over the seven different layers (Figure 9C). Percentage changes in model resistivities were in all seven layers at ERT1 and ERT3 negative, indicating profile‐wide decreasing resistivity values between the two campaigns in 2019 and 2022. At profile ERT2, only the uppermost five layers (down to 10 m) show a profile‐wide decrease in resistivity values. At the lowest two layers down to 15.8 m, both arithmetic mean and median increased during the 3‐year period.

### 4.3 Subsurface conditions based on direct observations

Insightful observations related to seasonal and perennial snow as well as to the subsurface conditions at the Hochtor area have been documented by the chief engineer of the Großglockner high alpine road.^15^ In his book on the history of the construction of this road, Wallack describes the existence of long‐lasting to perennial snow patches on both sides of the mountain pass in the 1930s. According to him, the south‐facing snow patch in Figure 3A,B only melted completely in warm years, whereas in cold years it stayed perennial. North of the pass there was the extensive snowfield of the ‘Obere Wintergrube’ (German for upper winter pit), which reached down to the northern tunnel portal and melted completely only at the tunnel portal site at the end of August every year. Today, small parts of this former extensive snowfield still exist, sometimes even perennially and are used by tourists in summer for recreation purposes.

**FIGURE 10:**
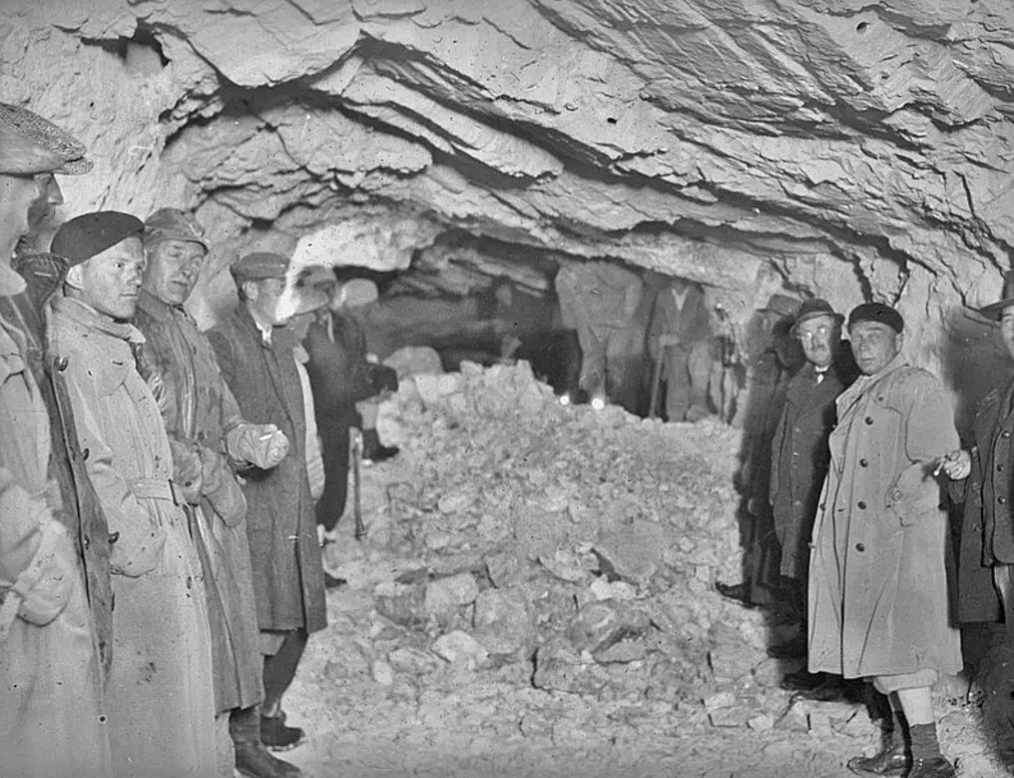
Tunnel breakthrough at the Hochtor tunnel in November 1933. The breakthrough was on 14 November 1933 about 70 m below the elevation of the Hochtor pass. The photo was taken soon afterwards. Note the dry‐looking conditions despite the fissured rock in the tunnel, suggesting either permafrost or dry mountain conditions. Photograph kindly provided by Archive GROHAG.

Construction of the Hochtor tunnel started with the first excavation works on 13 September 1933.^15^ It took 10 days to secure both tunnel portals because of thick periglacial slope sediments on both sides. On the second day of excavation works at the southern portal, the Roman Hercules statuette mentioned above was found at 1 m below the surface. The actual tunnel construction started on 23 September 1933, bedrock tunnelling was initiated on 1 October 1933 and the tunnel breakthrough occurred on 14 November 1933.^15^ Figure 10 gives an impression of this important event with people flanking the pile of rock in the centre. From a permafrost point of view, the observations by Wallack are of interest as he describes no problems with groundwater entering the tunnel. Furthermore, the picture shows that the drilled rock seems to be cleft rich, and thus groundwater might potentially infiltrate rather easily. However, the picture suggests dry and cold conditions inside the mountain and therefore might be interpreted as a permafrost environment. Even though the photo was taken in November, seasonal frost cannot be considered the main reason for dry conditions inside the mountain.

Observations during archaeological excavations accomplished in summer 1995 at the pass location gave insight into the vertical soil and sediment structure at the Hochtor area. The location and extent of the altogether six archaeological excavation sites located near the ERT profiles are drawn in Figure 4B based on photographs by Harl^4^ and on map sketches by Kastler.^3^ Harl^4^ describes that at all excavation sites at the Hochtor pass, only a thin layer of sediments and organic material covers the underlying bedrock. This bedrock was characterised by Harl^4^ as very soft and thin‐layered slatelike, describing the phyllitic character of the rock. The depth of excavations in 1995 was in the order of up to 50 cm as judged from the photographs presented by Harl.^4^ This implies that periglacial slope processes are possible to act only at the excavation sites at the uppermost decimetre and that the active layer exceeded at least 50 cm in summer 1995.

### 4.4 Long‐term evolution of ground thermal conditions

**FIGURE 11:**
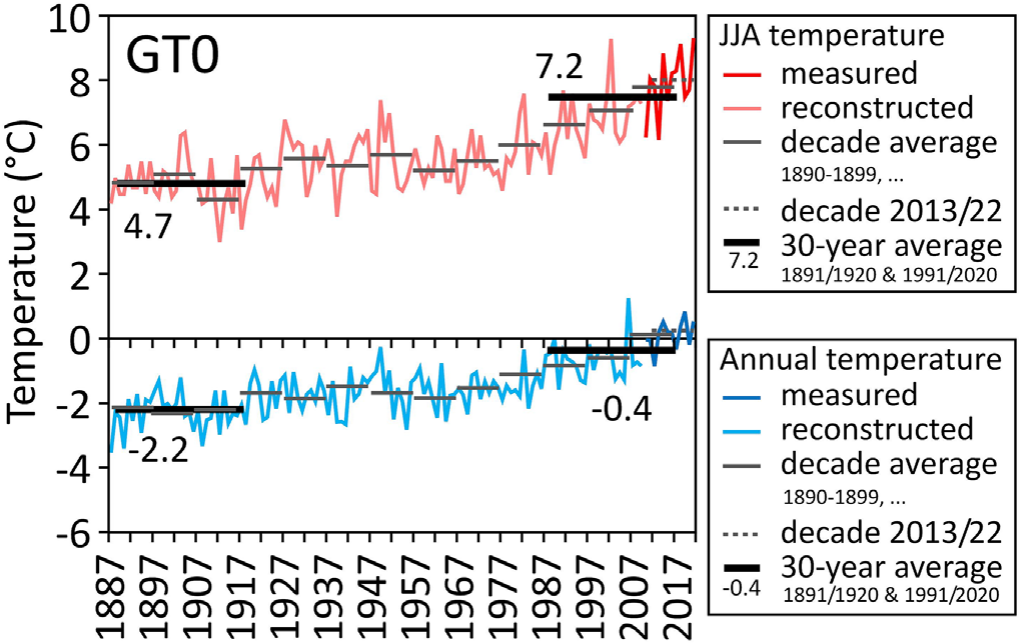
Measured (2010–2022) and reconstructed (1887–2009) evolution of the mean annual ground temperature and the mean summer [June, July and August (JJA)] ground temperature in the period 1887 to 2022. Average decade values starting with 1890–1899 (etc.), average of the last decade with data (2013–2022) and 30‐year average are plotted additionally. Reconstruction based on long‐term air temperature data from the nearby Sonnblick meteorological station (see text for details).

As GT measurements at the Hochtor pass indicate little influence of the seasonal snow cover at the GT site (Table 4), it was feasible to correlate the Hochtor GT data (GT0) with the Sonnblick air temperature data for the period 2010 to 2022. Correlation analyses between the seasonal and annual GT0 data with the respective Sonnblick data revealed significant correlations at the 0.01 level for all four seasons and the annual data (Pearson correlations: annual value r = 0.76; seasonal values: MAM 0.81, JJA 0.92, SON 0.79, DJF 0.79). In the following, we will focus on the reconstruction of the annual and summer temperatures at the GT site. The average differences of the mean values for Hochtor and Sonnblick during the period 2010–2022 were 4.14°C (SD 0.30) for annual and 4.58°C (SD 0.39) for summer temperatures. These differences were used to estimate GT conditions at the Hochtor site back to 1887 (Figure 11), when the recording of the Sonnblick values started.

Summer temperatures have increased much more distinct than the annual mean values since 1887 (Figure 11). The three warmest monitoring years at Hochtor since 1887 were 2006/2007 (01 September 2006–31 August 2007) with 1.25°C MAGT, estimated, followed by 2019/2020 (0.83°C) and 2021/2022 (0.53°C), both measured. Furthermore, the three warmest summers since 1887 at this site were 2022 (9.31°C; measured), 2003 (9.28°C; estimated) and 2019 (9.13°C; measured). The decade average values (1890–1899, etc., as well as the decade 2013–2022) for both summer and annual ground surface temperatures are also depicted in Figure 11. These average values reveal rather stable to weak warming conditions until the 1970s with a distinct warming trend in the past four decades. Summer warming is more pronounced than the increase in annual values. The difference in the decade average of 1890–1899 and 2013–2022 is +2.3°C for annual values and +3.1°C for summer temperatures. Only during the most recent decade was the average at Hochtor above 0°C. Finally, the mean annual temperature of the 30‐year reference period 1991–2020 was 1.8°C higher compared to 1891–1920. The difference in the average temperature of JJA for the same two 30‐year periods is even 2.5°C.

## 5 DISCUSSION

### 5.1 Permafrost versus seasonal frost

GT monitoring in the period 2010–2022 indicates that the Hochtor site GT (Figures 3C and 4B) at 2,582.5 m asl is characterised by an MAGT of around 0°C at the surface but also at depths down to (at least) 60 cm. All three GT sensors are affected by seasonal frost with annual temperature amplitudes of 17°C at the surface and 13.2°C at 60 cm depth. Based on the extrapolation of monthly temperature profiles to greater depths (Figure 6), according to the approach by van Everdingen,^47^ we estimated the thickness of annual thawing and annual freezing for the 12 monitoring years 2010/2011 to 2021/2022. With this, we showed that in most of the last 12 years, the calculated DOT exceeded the depth of seasonal freezing. During the last years, annual thawing thicknesses surpassed almost always annual freezing. These unfavourable recent climatic conditions for the cryosphere are also indicated by the substantially decreasing extent of late‐lying (at the south‐facing slope; Figure 3A,B) and perennial (at the north‐facing slope near the tunnel portal) snow patches at both sides of the mountain pass if the historic descriptions and photographs from the 1930s by Wallack^15^ are compared with our own observations in the period 2010–2022.

Results of the geoelectric profile measurements in 2022 imply that permafrost is present along all three ERT profiles measured. Kneisel and Hauck^42^ summarised that resistivity values for frozen sediments may range from 1 k Ω.m to 1 Mio. Ω.m depending mainly on ice content. The resistivities of unfrozen phyllites and schists at the ERT measurement sites at the Hochtor site are not known. However, based on literature, schist might have resistivity values from between 100 and 10 k Ω.m, mostly around 3 k Ω.m.^49^, ^50^ Resistivities in crystalline rocks with foliation such as schist depend also on the direction of measurement relative to the foliation or texture. Finally, the range of resistivity values for most materials can also be explained by the varying water or pore air content with wet = low value and dry = high value.^49^

The nature of overlapping resistivities for different materials and the influence of pore air make the interpretation sometimes difficult.^42^ At the Hochtor site we know, nevertheless, from the archaeological excavations in 1995 (Figure 4B) that the soil and sediment layer covering bedrock at the flatter part of the Hochtor site, where ERT was mainly accomplished, is thin. This layer is only in the order of decimetres thick covering bedrock. Thus, changes in resistivities in the uppermost metres of a vertical profile at the Hochtor site must indicate changes in moisture, air or ice contents. A clear three‐layer structure with a low‐resistivity upper layer at the top (2–3 m), high‐resistivity values in a several‐metre‐thick middle layer (about 5–10 m) and low to medium high‐resistivity values below this layer (Figures 8 and 9) suggests a lenslike permafrost layer at all profiles in the Hochtor mountain pass area. Based on the resistivity pattern depicted in Figures 8 and 9, we consider a threshold value of 3 k Ω.m as plausible to differentiate between frozen and unfrozen phyllitic bedrock at this site. This is in accordance with Otto et al.,^51^ who interpreted resistivity values of up to 3 k Ω.m as unfrozen fine‐grained, phyllitic substrate. Median resistivity values in 2022 were in the range of 2,500 to 2,700 Ω.m at all three profiles, and maximum resistivities varied between 8,700 Ω.m (ERT3) and 70,300 Ω.m (ERT2). Therefore, we argue that ice‐poor permafrost existed in 2022 in all three profiles in the form of a 5 to 10 m thick lens.

### 5.2 Long‐term thermal changes since 1887

A distinct warming trend of ground thermal conditions since 1887 was reconstructed for the Hochtor site based on long‐term Sonnblick data and the significant correlation between air temperature and GT data in the overlapping data period from September 2010 to August 2022 (for annual data r = 0.76; for JJA data r = 0.92). Summer warming was much stronger compared to mean annual warming. The strongest warming in the Alps during the remaining part of the 21st century is projected for the summer season.^52^ Our reconstruction is based solely on long‐term air temperature data, however, and neglects any possible changes in the surface offset, which is the difference in temperature between the air and the ground surface.^53^ Commonly, the nival offset in winter is larger than the vegetation offset in summer,^54^ and therefore the MAAT is commonly lower than the mean annual GT at the surface. Based on our field observations and data since 2010 as well as on earlier descriptions by Wallack^15^ going back to the 1930s, we can assume that the wind‐exposed site GT was commonly snow free for most of the winter seasons. Therefore, the calculated ground surface warming of 1.8°C for the annual average and 2.5°C for the summer season between the two reference periods 1891–1920 and 1991–2020 seems plausible.

### 5.3 Thermal changes and impacts in the period 2010–2022

Continuous GT data since August 2010 until September 2022 offer insight into the ground thermal evolution of the Hochtor area. High interannual variability in annual values for all parameters may hide meaningful trends, and thus caution should be exercised when making trend statements. Therefore, one could determine that no significant trends were detected based on annual data (monitoring years) apart from the decreasing length of the seasonal snow cover (from 18 to 0) and an increase in the number of days with an MGT > 5°C (from 67 to 116).

The same caution must be applied to seasonal single‐year values. However, despite this limitation, a warming tendency exists for GTs at this site for the winter, autumn and, in particular, summer seasons. In contrast, a light tendency of cooling was quantified for spring for the period 2010–2022. In the long‐term (here 1887–2022), there is, nevertheless, a clear warming trend also in the spring temperatures, as shown by meteorological data of the nearby Sonnblick station. Because a distinct seasonal winter snow cover at the GT monitoring site is absent over the entire winter due to wind‐related snow redistribution from the ridge towards south, forming a cornice (Figure 3A,B), ground thermal changes directly mirror atmospheric changes at the Hochtor site. As averaging over several years helps quantify climatic changes (advised are 30 years^55^), we compared three time slices of 4 years each (September 2010/2008–2014; September 2014/2008–2018; September 2018/2008–2022). With this approach, we were able to reveal ground warming based on the parameters MGT (by 0.58°C), TDD (by +165), FDD (by −59), as well as an increase or decrease in the number of days with a mean temperature below or above certain thresholds (<−15°C, <−10°C, >5°C). This warming tendency is well in line with atmospheric warming measured at the nearby Sonnblick station (Figure 2; data source HISTALP database^2^). At Sonnblick, the averages of the MAATs for the same three 4‐year periods increased from −4.33°C (2010/2014) to −4.08°C (2014/2018) and finally to −3.98°C (2018/2022), thus by +0.35°C, which is 60% of the warming at the Hochtor site. These observations are consistent with ground surface and air temperature changes in the Niedere Tauern Range^26^ or the Swiss Alps.^56^

Estimations of the annual freezing and thawing depths between 2010 and 2022 support the measured GT data. A dominance of annual freezing versus annual thawing (thus permafrost‐favourable conditions) was quantified for most of the first 6 years of this period, whereas in the latter 6 years, annual thawing exceeded annual freezing (thus permafrost‐unfavourable conditions). Although a linear extrapolation of freezing and thawing depths is a very simplified assumption,^47^, ^48^ this analysis suggests that during the observations period, the permafrost situation at the Hochtor site changed. The alteration was from an active permafrost site to an inactive one with a (presumably thin) supra‐permafrost talik zone in between the seasonally thawing and freezing top layer and the permafrost. Any permafrost that occurs at present at this mountain pass was affected during most of the last years by degradation in terms of warming and partly thawing of permafrost‐related ground ice.

### 5.4 Significance of repeated ERT measurements

A further interesting insight into the impact of ongoing ground thermal warming on permafrost compares the ERT measurements at identical profiles in 2019 and 2022. By repeating initial profile measurements after a period of 3 years, we detected changes in the ground conditions and in the evolution of permafrost. Repeating ERT measurements over a multiannual period is a powerful approach to monitoring long‐term permafrost evolution. Hauck^57^ successfully initiated such ground monitoring in permafrost terrain at Schilthorn, Swiss Alps, to study the permafrost evolution over monthly to seasonal time scales. Later, ERT monitoring was automated using different approaches in the Swiss^58^ and Austrian Alps.^59^ Mollaret et al.^60^ proved that permanently operating ERT monitoring [using the term electrical resistivity tomography monitoring (ERTM)] at six sites in the Swiss Alps is efficient in documenting the degradation of permafrost in Alpine regions. ERT monitoring is even part of the Swiss Permafrost Monitoring Network activities with fixed installed profiles and measurements once a year at the end of summer.^56^

Drawbacks in repeating previous ERT surveys could be different measurement devices used during the initial and the repeated campaign or finding again the initial course of a profile or even the position of the individual electrodes (commonly the steel rods). Buckel et al.^61^ repeated ‘historical’ ERT surveys after periods of 10 to 16 years in the Swiss and Austrian Alps. The accuracy of their historical and more recent measured location data is within the range of a standard GPS device, which is 5–10 m horizontally.^61^ Furthermore, finding the location of historical ERT profiles might be accomplished by refinding distinct boulders visible in earlier orthophotographs or other sources.^61^ In our study, we were able to exclude the problem of localising the initial profiles or because of the usage of different instruments in a further field campaign.^59^ We carried out our ERT measurements in both years at the same profiles (based on the GNSS data), with the same device (Geotom‐2D System) and with the same set‐up (4 m spacing, 25 spike electrodes). One minor drawback might be the displacement of the spike electrode locations over a 3‐year period, as the Hochtor area is affected by solifluction displacing archaeological artifacts^32^ and forming distinct solifluction terraces and lobes (Figures 3 and 4). Solifluction rate data are not known from the Hochtor site. As judged from field‐based measurements at a nearby site (surface velocity rates of 3.5 cm/year^26^), we can assume a maximum relative misplacement of individual electrode positions between 2019 and 2022 in the order of a maximum of 10 cm. This minor change in the position is considered irrelevant if compared to, for instance, those of active rock glaciers.^61^

Keeping in mind these minor drawbacks in the comparison between the 2019 and 2022 data, we can conclude that our quantified time‐lapse changes in resistivities mirror pure alterations in the subsurface. On average over the entire profile length and the entire depth (median of all values), overall resistivities decreased between 2019 and 2022 by 3.9% to 5.2% per year. This pattern underlines once again permafrost degradation in terms of the thawing of previously frozen ground.

Resistivity changes were also quantified for the seven different depth layers, in our case starting at 1 m and ending at 15.8 m depth (ERT with 96 m length). The lowering of the median values between 2019 and 2022 was calculated for all seven layers at ERT1 and ERT3. At ERT2, the median values increased at layers six (12.7 m) and seven (15.8 m). This increase in the resistivities at greater depth seems to be inconsistent with the assumed permafrost degradation along the entire profile. A possible interpretation of the increased values of these two layers (but also generally in the profiles; Figure 8D) could be a higher proportion of air voids in the subsurface in 2022 compared to 2019, which is due to drier conditions in the last year. The measurement in 2019 was accomplished on 17 August, whereas the measurement in 2022 was carried out 26 calendar days later on 12 September.

As summer 2022 was exceptionally warm and dry, one could expect at ERT profiles in fine‐grained, void‐poor substrates in non‐permafrost environments a decrease in the near‐surface soil humidity and thus an increase in air‐filled voids over time. This implies an increase in resistivity values during summer. In contrast, in permafrost areas, the process of permafrost degradation by the melting of interstitial ice might lead to a decrease in the resistivity values, as ice in the voids is replaced by water or even air. In the case of the dominance of the air, resistivity values might rise again due to the poor conductivity of air.^42^ Dryer conditions at the Hochtor site in 2022 compared to 2019 can be assumed, considering the precipitation data from the nearby Sonnblick station (Figure 2). At this station, the precipitation sum in the 30 days before the measurements was 174 mm in 2019 whereas only 132 mm in 2022 (data GeoSphere Austria).

It could also not be ruled out that a fracture in the rock structure (further) opened between 2019 and 2022 around profilemeter 60 of ERT1 and ERT2. Thus, the proportion of air‐filled pore space at about 60 to 65 m from the southern beginning of the profile increased in both ERT profiles, causing higher resistivity values at this location in 2022 compared to 3 years earlier. The indicated oblique linear structure of the area with increasing resistance value in Figure 8D could be an indication of this hypothesis. Because the bedrock is rich in brittle structures, such a scenario is conceivable.

### 5.5 Implications in the potential weathering and solifluction by frost action

Present rates of solifluction or weathering based on frost action at the Hochtor site are unknown. Based on solifluction monitoring results on a nearby, lower‐elevated (2,247 m asl) turf‐banked lobe,^26^ annual surface velocities in the order of 3.5 cm/year seem to be a minimum for many of the solifluction features at the Hochtor site, as many of them are stone‐banked lobes, which are more exposed to the four different types of solifluction processes (as defined by Matsuoka^25^).

Two common models for frost weathering are generally distinguished. These are the volumetric‐expansion or hydro‐fracturing model (M1) when in situ water expands by 9% in volume due to freezing^62^ and the segregation‐ice model (M2) where water potentially migrates within freezing or frozen ground, supplying progressive growth of ice lenses.^63^ Based on our GT data, we derived frost weathering–related parameters such as days with FTCs (for M1) or days within a predefined FCW (for M2). Regarding M1, we observed a distinct decrease in the number of FTC using mean values of 4 years from 70 days in 2010/2011–2013/2014 to 50 days in 2018/2019–2021/2022. Neglecting rock moisture and rock properties in determining rock weathering,^64^ we assume that hydro‐fracturing at the Hochtor site decreased significantly during the 12‐year monitoring period. Regarding M2, the days in the frost FCW have decreased for both value ranges (−3°C to −6°C and −3°C to −8°C). For FCW36 this decrease was −56% (from 24 in 2010/2011–2013/2014 to 11 in 2018/2019–2021/2022), whereas for FCW38 the same value was −39% (from 59 in 2010/2011–2013/2014 to 36 in 2018/2019–2021/2022). Thus, we accept a substantial decrease in frost‐related weathering at the Hochtor site since at least 2010.

### 5.6 The effect of the summer heatwave in 2022 at the Hochtor site

Based on air temperature data from the nearby Sonnblick site and our measured and reconstructed GT (Figure 11), we can assume that the most severe heatwave hitting the Hochtor area in the period of 1887–2022 occurred in 2003 with a summer temperature anomaly of +2.1°C above the reference period 1991–2020 (Figure 1B). Second in place is 2019 (anomaly +2.0°C), followed by 2022 and 2015, both of which show the same anomalies averaged over the summer (+1.6°C). The measured GT data series at Hochtor do not go back to 2003, but the following ranking considers the summers 2010 to 2022: 2022, 9.3°C; 2019, 9.1°C; 2015, 8.8°C. The reconstructed summer temperature for 2003 is, nevertheless, also 9.3°C. This ranking differs slightly from that of Sonnblick (excluding 2003), but it shows well that the summers of 2003, 2015, 2019 and 2022 (Figure 1B) were all exceptionally warm and the warmest during the entire period 1887–2022.

As both summers with ERT data from the Hochtor site are in the top three regarding ground and air temperatures, we can assume, in general, similar ground thermal conditions during the summers of 2019 and 2022. However, the winters preceding warm summers must also be considered regarding thermal and related snow cover conditions. For instance, the comparison of the winter temperature and snow data at Sonnblick based on HISTALP for the winters 2002/2003 and 2021/2022 shows clear differences. Overall, winter 2002/2003 and spring 2003 were colder than the same seasons in 2021/2022 but had, on average, about twice as much snow in terms of maximum daily snow depths. This indicates a much better air temperature–soil temperature coupling in 2021/2022 compared to 2002/2003 and thus more efficient ground cooling. Because the Hochtor field site generally has little snow due to its wind‐prone pass location, the described effect of the snow‐related increased or reduced air‐ground thermal coupling probably plays a minor role. Because of the negligible snow effect at the Hochtor site, ground surface cooling in 2021/2022 was presumably less effective at this site compared to 2002/2003.

The changes in the resistivities we quantified between 2019 and 2022 must be seen as a long‐term signal of permafrost degradation and not as the single effect of the most recent summer heatwave of 2022. It is most likely that this long‐term degradation of permafrost will continue at the Hochtor site and elsewhere in the Alps. This assumption is also indicated by a thermal delaying effect revealed by borehole data^48^, ^56^ and by the predicted 21st‐century alpine climate change.^52^ The interpreted downward degradation of permafrost at the Hochtor site develops commonly when the maximum depth of seasonal thawing exceeds the maximum depth of seasonal freezing, resulting in the formation of the supra‐permafrost talik. This talik disconnects the permafrost from the seasonal frost layer, further enhancing permafrost degradation.^65^ Based on our observed development of the GT and the resistivity values, it can be assumed that at least the near‐surface permafrost at the Hochtor will soon be history. Whether deeper‐lying permafrost is still to be expected at the Hochtor, as one might guess from historical photos from the 1930s, is still an open question.

## 6 CONCLUSIONS

Using a combination of direct GT data (2010–2022), repeated geophysical measurements (2019 and 2022) and auxiliary data dating back to 1887 (instrumental data) or even Roman times (knowledge of subterranean rock structures and ground material displacement based on archaeological finds, excavations and tunnel drilling), we draw the following conclusions from this study.

A general three‐layer structure with a 2–3 m thick low‐resistivity top layer, a 5–10 m thick high‐resistivity central layer and low to medium high‐resistivity values below these two layers down to 16 m was quantified for three ERT profiles measured at the flat Hochtor pass area. The middle layer is interpreted as a lenslike, ice‐poor permafrost layer, which existed in 2019 and 2022. This conclusion is consistent with the measured ground thermal conditions and interpretations based on archaeological excavations, which also indicate marginal permafrost to seasonal frost conditions at the Hochtor site.

A clear warming trend was revealed at the Hochtor pass for the summer season and at least a warming tendency for the winter and autumn seasons in the period 2010–2022. In contrast, a light tendency of cooling is implied for the spring season. On an annual timescale and averaged over three 4‐year intervals, the MGT significantly increased, whereas the number of FDDs significantly decreased.

By accomplishing repeated ERT measurements after a period of 3 years, we were able to detect distinct changes in the resistivity pattern of all three profiles. As we carried out our ERT measurements in both years at the same profiles (retrieval of initial profiles with GNSS), with the same geoelectrical device and with the same measuring arrangement (spacing, length of profile, type of electrodes), we observed a decreasing rate in mean annual resistivity at the three profiles of 3.9% to 5.2% per year averaged over the entire profile. We interpret this as permafrost degradation.

During the observation period 2010 to 2022, the Hochtor site presumably changed at least at the uppermost 16 m from an active permafrost site to an inactive one with a supra‐permafrost talik zone in between the seasonally thawing and freezing top layer and the permafrost. Permafrost at greater depth (<16 m) may still be present at the Hochtor site as photographs from a historical tunnel project possibly suggest, but this remains to be investigated in the future.

The calculated ground surface warming between two climate reference periods (1891–1920, 1991–2020) suggest an increase of the mean annual ground surface temperature by 1.8°C (from −2.2°C to −0.4°C) and of the mean summer ground surface temperature by 2.5°C (from 4.7°C to 7.2°C). Summer warming was stronger than annual warming, which matches with previous works and future scenarios. On a decade scale, only during the most recent decade (2013–2022), the average ground surface temperature was above 0°C at the Hochtor site.

Unrelated to the general model for frost weathering used (hydro‐fracturing or frost‐cracking), a significant decrease in frost‐related weathering at the Hochtor site since at least 2010 is most likely. Changes in FTCs and in the frost behaviour presumably impacted the weathering behaviour and solifluction rates of the widespread solifluction lobes and terraces at the Hochtor site. However, understanding of solifluction processes and their changes at the Hochtor site is presently poor and remains to be investigated in the future.

The summers of 2003, 2015, 2019 and 2022 were the four warmest ones during the entire 135‐year period 1887–2022 with indirect (reconstructed based on correlation with nearby air temperature data) and direct GT data at the Hochtor site. Permafrost at Hochtor was affected during most of the last years by the warming and partly thawing of ground ice. The changes in the resistivities we observed between 2019 and 2022 must be seen as a long‐term signal of permafrost degradation and not as the single effect of the summer heatwave of 2022. However, heatwaves amplify this warming trend. As we will face a warmer climate during the 21st century, we argue that at least the near‐surface permafrost at the Hochtor site will soon belong to the past.

## CONFLICT OF INTEREST STATEMENT

The authors declare no conflicts of interest.

## Data Availability

The data that support the findings of this study are available from the corresponding author upon reasonable request.
